# Laser interstitial thermal therapy for brain metastases

**DOI:** 10.1093/noajnl/vdab128

**Published:** 2021-11-27

**Authors:** Ethan S Srinivasan, Matthew M Grabowski, Brian V Nahed, Gene H Barnett, Peter E Fecci

**Affiliations:** 1 School of Medicine, Duke University, Durham, North Carolina, USA; 2 Department of Neurosurgery, Cleveland Clinic & Case Comprehensive Cancer Center, Cleveland, Ohio, USA; 3 Department of Neurosurgery, Massachusetts General Hospital, Boston, Massachusetts, USA; 4 Department of Neurosurgery, Duke University Medical Center, Durham, North Carolina, USA

**Keywords:** brain metastases, LITT, radiation necrosis, recurrent metastases

## Abstract

Laser interstitial thermal therapy (LITT) is a minimally invasive treatment for intracranial lesions entailing thermal ablation via a stereotactically placed laser probe. In metastatic disease, it has shown the most promise in the treatment of radiographically progressive lesions after initial stereotactic radiosurgery, whether due to recurrent metastatic disease or radiation necrosis. LITT has been demonstrated to provide clinical benefit in both cases, as discussed in the review below. With its minimal surgical footprint and short recovery period, LITT is further advantaged for patients who are otherwise high-risk surgical candidates or with lesions in difficult to access locations. Exploration of the current data on its use in metastatic disease will allow for a better understanding of the indications, benefits, and future directions of LITT for these patients.

Metastatic brain tumors make up more than 50% of all intracranial tumors and affect an estimated 10%–20% of solid tumor patients, with autopsy studies indicating that number could be even higher.^1–4^ Today, the typical standard of care for newly diagnosed metastases to the brain is stereotactic radiotherapy, with surgical resection offered for cases with significant mass effect or cerebrospinal fluid obstruction.^5–7^ In this context, laser interstitial thermal therapy (LITT) emerged as a minimally invasive surgical treatment option for tumors that were either less accessible via craniotomy, or for which craniotomy had a narrower therapeutic window.

LITT entails thermal ablation via a stereotactically placed laser probe. Its role in metastatic disease has been investigated over the past decade, with the most promising results found in treatment of lesions progressing after stereotactic radiosurgery (SRS) due to either recurrent metastatic disease or radiation necrosis (RN). The highly targeted SRS yields an overall local control rate for brain metastases of 70%–90% at 1 year, with 9%–14% of these patients developing RN.^8–10^ This remaining population, those with either radioresistant progressive intracranial disease or with RN, stand to benefit most from LITT given its utility in both progressive etiologies. Furthermore, the 2 pathologies appear radiographically similar on imaging studies, as shown in patient MRI examples of Figure 1, and LITT permits intraoperative histological diagnosis that can guide additional treatment decisions. As targeted and immunotherapeutic options for systemic disease continue to advance, it is likely that more and more patients will survive to this point.^5^Table 1 provides a summation of the LITT studies to date in this patient cohort. With no definitive protocols to date for management of these cases, understanding the role of LITT and factors that define appropriate patient selection are valuable tools in the neuro-oncologic armament.

**Table 1.** Existing Studies on the Use of Laser Interstitial Thermal Therapy (LITT) for Radiographically Progressive Metastatic Lesions After Stereotactic Radiosurgery (SRS)

Author (Year)	Patient Numbers	Diagnostic Modality	Pretreatment Volume (cc)	Follow-up (Months)	Local Control Rate (%)		Overall Survival (%)	
					6 Months	12 Months	6 Months	12 Months
Carpentier (2011)^11^	Total: 7	MRI		13.3	66.9	58.3	83.3	66.6
	BM: 7	MRI		13.3	66.9	58.3	83.3	66.6
	RN: 0	MRI						
Ali (2016)^12^	Total: 23		4.9 (0.4–28.9)	4.7 (range 2.1–26.5)	72.6	56		
	BM: 23		4.9 (0.4–28.9)	4.7 (range 2.1–26.5)	72.6	56		
	RN: 0							
Smith (2016)^13^	Total: 6	Biopsy			56.5	40.8	66.7	66.7
	BM: 0	Biopsy						
	RN: 6	Biopsy			56.5	40.8	66.7	66.7
Ahluwalia (2018)^14^	Total: 42	Biopsy	6.4 ± 6.7				72.2	
	BM: 20	Biopsy	7.1 ± 8.7		54		64.5	
	RN: 19	Biopsy	5.5 ± 3.9		100		82.1	
Chaunzwa (2018)^15^	Total: 30	MRI	7.6 (0.6–38.9)	5.9 (range 1–31)	92.9		52.3	26.1
	BM: 5	MRI						
	RN: 19	MRI						
Salehi (2018)^16^	Total: 24	NA	7.32 (1.00–24.59)	32.26 (range 7.2–46.73)			76.3	59.4
	BM: NA							
	RN: NA							
Hong (2019)^17^	Total: 34	Biopsy	4.1		75.6	72.2	79.4	69.0
	BM: 16	Biopsy						
	RN: 18	Biopsy						
Bastos (2020)^18^	Total: 61	MRI	4.02 (0.2–26.3)	7 (IQR 4–21.5)	69.6	59.4	80.6	65.8
	BM: 34	MRI			64.1	50		
	RN: 27	MRI			91.5	80		
Kim (2020)^19^	Total: 92						82.0	73.7
	BM: 43							
	RN: 34							
Luther (2020)^20^	Total: 20	Biopsy	8.5 (0.9–31.7)		67.2	67.2	87.8 (59.5–96.7)	71.9 (40.6–88.6)
	BM: 0							
	RN: 20	Biopsy	8.5 (0.9–31.7)		67.2	67.2	87.8 (59.5–96.7)	71.9 (40.6–88.6)
Shah (2020)^21^	Total: 36	Biopsy	4.3 (0.6–28.0)	7.2 (IQR 3.0–16.9)	81.9	77.4		
	BM: 36	Biopsy	4.3 (0.6–28.0)	7.2 (IQR 3.0–16.9)	81.9	77.4		
	RN: 0							
Sujijantarat (2020)^22^	Total: 25	Biopsy	2.2 (0.3–12.6)	24 (IQR 3.0–16.9)	96		96	84
	BM: 0							
	RN: 25	Biopsy	2.2 (0.3–12.6)	24 (IQR 3.0–16.9)	96		96	84
Kaye (2020)^23^	Total: 70	MRI		12 (range 0.5–78.1)			72.4	51.8
	BM: NA							
	RN: NA							
Hernandez (2019)^24^	Total: 45	MRI	3.4 (0.24–10.8)	10.5 (1.9–59.1)	89.8	84.7		
	BM: NA							
	RN: NA							

Adapted from Chen et al.^25^

**Figure 1.**
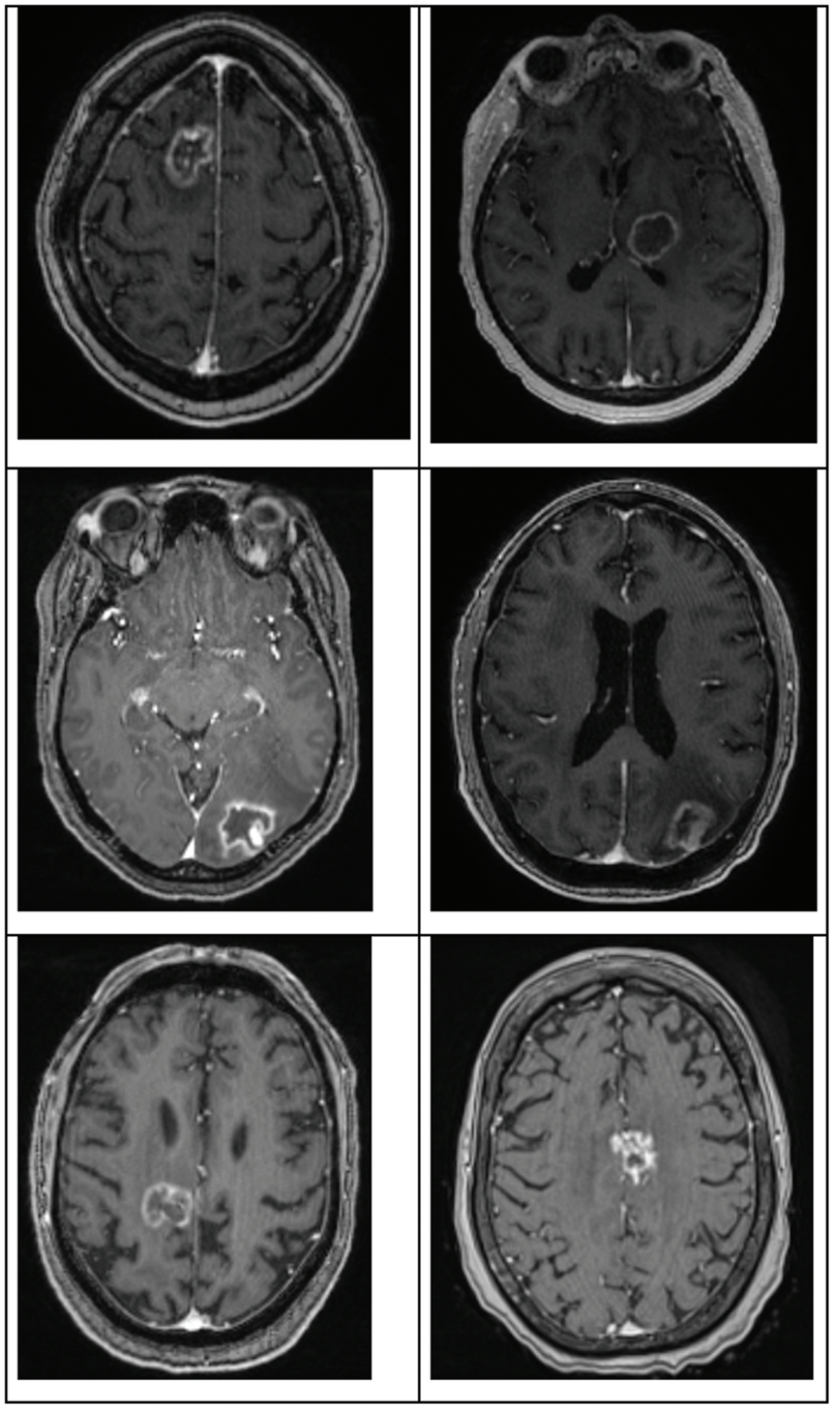
Clinical T1-weighted magnetic resonance imaging with contrast of radiographically progressive poststereotactic radiosurgery metastatic lesions in human patients. Each was subsequently treated with LITT and lesions on the left were proven to be radiation necrosis while lesions on the right were recurrent metastatic disease. LITT, laser interstitial thermal therapy.

## LITT for Recurrent Metastatic Disease

**Figure 2.**
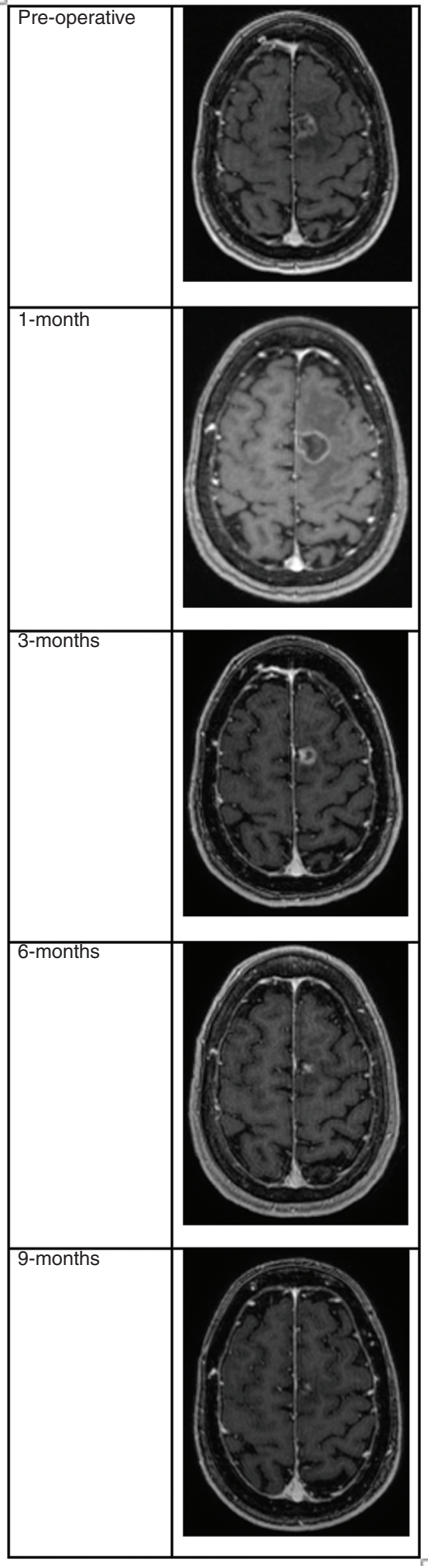
T1-weighted magnetic resonance imaging of a patient treated for recurrent metastatic lung cancer. Imaging progression shows typical evolution of effective laser interstitial thermal therapy (LITT) ablation with initial contrast-enhancing thin-rimmed expansion followed by contraction over the following months.

LITT for recurrent metastatic disease was first described in 2008 by Carpentier et al., with their report of its use in 4 patients. Each had previously received systemic and radiation therapy before presenting with tumor progression to a size <3 cm in the cerebral hemispheres. This initial study demonstrated the feasibility of the approach, with all 4 experiencing minimal discomfort, discharged within 14 h of the procedure, and showing subsequent radiographic response at the ablation site.^26^ Similar to LITT in other contexts, the lesions showed a characteristic post-treatment evolution of an initial thin-rimmed expansion, followed by contraction, as demonstrated in a representative case in Figure 2. This initial expansion is an important sequela of LITT treatment with clinical consequences limiting patient candidacy. The subsequent studies in larger populations supported the clinical efficacy and safety of the procedure, with 6-month local control rates of 67%–73% specifically associated with the extent of ablative lesion coverage.^11,12^

Further research in this patient population has been limited to some degree, as many studies combine recurrent metastatic disease with RN or primary tumors for their analyses.^16,23^ This heterogeneity of data can be qualitatively observed in Table 1, which divides LITT studies by etiology where possible and highlights the varied methodologies. Results from studies specifically reporting outcomes in the recurrent metastatic disease population have been encouraging with 6- and 12-month local control rates of ranging from 54% to 81.9% and 50% to 77.4%, respectively.^11,12,18,21,27^ A recent meta-analysis sought to consolidate these results as Chen et al. identified 9 relevant studies and analyzed the data for recurrent metastases alone. Their work posited overall local control rates of 67.9% at 6 months and 59.9% at 12 months, and overall survival (OS) rates of 69.2% and 66.5%.^24^ While these studies in specific and aggregate present a compelling case for LITT, an important qualification to note is that the described patients were all initially chosen as appropriate candidates for the therapy rather than at random.

Regarding factors associated with good outcomes, Salehi et al. redemonstrated the importance of complete lesion ablation as coverage >97% was associated with improved progression-free survival (PFS), though their study did not fully differentiate between recurrent tumor and RN.^16^ When compared to alternative treatments, Hong et al. found no difference in local control or OS relative to open resection out to 2 years (43.8%–44.4% and 48.6%–40.2%, respectively). In the same study, LITT was also shown to result in significantly shorter hospital stays, though craniotomy provided higher rates of resolution for preoperative deficits.^17^ These differences highlight the importance of patient selection, as each treatment option offers advantages and disadvantages that should be tailored to individual patient characteristics and values. Further evaluation of LITT’s integration as a combination therapy with SRS are essential next steps in optimizing the use of LITT for this pathology.

**Figure 3.**
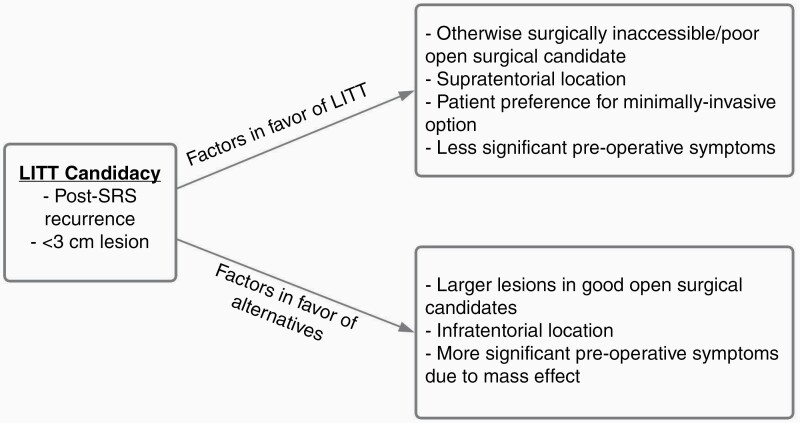
Flowsheet describing the general algorithm for identifying patients who would benefit from LITT to their metastatic brain lesion. LITT, laser interstitial thermal therapy; SRS, stereotactic radiosurgery.

Taken together, these results indicate that LITT is both a safe and effective option for the treatment of recurrent metastatic disease in the correct patient population that would benefit from the minimally invasive approach to cytoreduction. Given the data, the ideal patient is 1 with a smaller (generally <3 cm), supratentorial lesion that is either superficial or deep-seated, who may not be a candidate for open resection or prefers the shorter hospital stay and low morbidity associated with the LITT procedure.^19,28–30^ Those with larger lesions are not typically amenable to the treatment, with particular concern for the initial postoperative swelling that can lead to serious complications if too extensive.^31,32^ An overarching takeaway from the results thus far proposes that LITT is an effective therapy for recurrent brain metastases when administered to a selected population based on clinical and radiographic features as well as patient preferences. In practice, the algorithm for this decision continues to evolve but a general framework is presented in Figure 3.

## LITT for RN After SRS

Few treatments have revolutionized the treatment of brain metastases like SRS. However, approximately 9%–14% of patients will develop RN—a proliferative, inflammatory process that can beget significant morbidity and impaired quality of life as a result of vasogenic edema and mass effect.^33^ RN usually manifests itself anywhere from 3 months to 3 years after SRS, and has been correlated to a number of clinical variables, most notably those related to SRS treatment characteristics.^34,35^ While self-limiting in up to 50% of cases, RN after SRS for brain metastases presents a unique challenge for clinicians for a few reasons: (1) noninvasive diagnostic studies have limited utility in distinguishing between tumor recurrence and RN, (2) each pathology has its own opposing noninvasive treatment modality, and (3) once accurately diagnosed, RN can potentially require prolonged steroids, bevacizumab, or even open surgical therapy, with the respective negative sequelae of each treatment (including deleterious impact on important primary disease therapies such as immunotherapy).^22,36,37^ Because of the aforementioned reasons, LITT was put forth as a minimally invasive, definitive treatment modality for RN.

**Figure 4.**
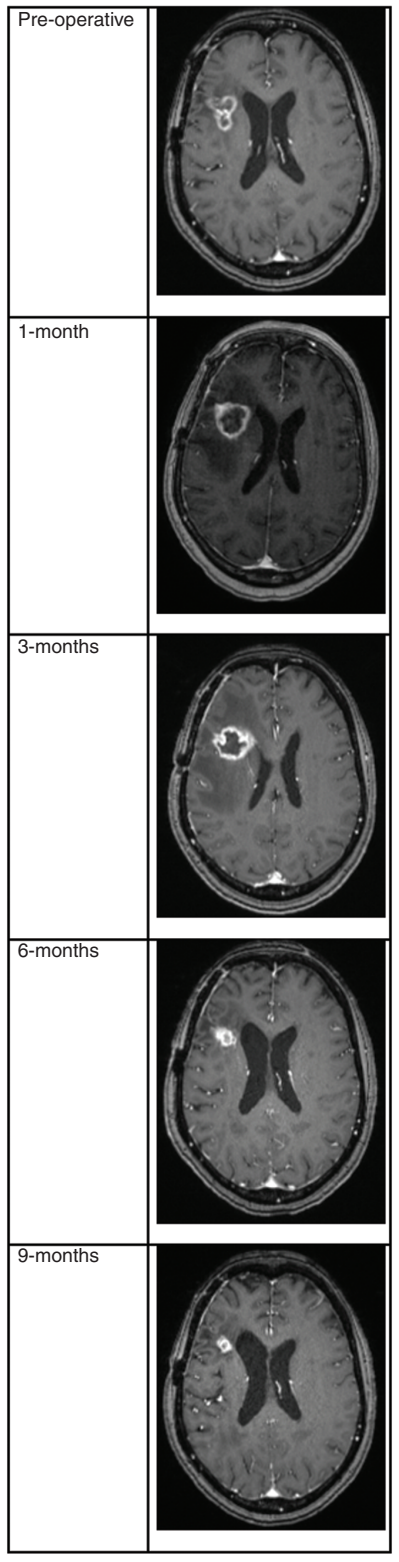
T1-weighted magnetic resonance imaging of a patient treated for radiation necrosis after SRS for metastatic lung adenocarcinoma. Imaging progression shows typical evolution of effective laser interstitial thermal therapy (LITT) ablation with initial contrast-enhancing thin-rimmed expansion followed by contraction over the following months. SRS, stereotactic radiosurgery.

LITT was first described for the treatment of RN in 2012, and since then has gained more widespread use for this indication after initial studies reporting on PFS. Figure 4 demonstrates the characteristic evolution of an RN lesion over the first 9 months after LITT treatment. In a retrospective study with long-term follow-up, Smith et al. reported a median PFS of 11.4 months in a group of patients treated with LITT for biopsy-proven RN.^13^ In the first prospective trial of LITT for brain metastases at 6 centers in the United States (LAASR Trial), the RN group had a PFS of 100% at 12 weeks and 91% at 6 months, while OS was 100% at 12 weeks and 82.1% at 6 months.^27^ In Shah et al.’s series of 20 patients with biopsy-proven RN, median time to recurrence was not reached, with 75% of symptomatic patients having local control at 1 year.^38^ Hernandez et al. reported a local control rate of 83.1% at a median follow-up of 44.6 weeks in 59 treated patients, with LITT failure occurring at a median of 18.8 weeks post-LITT (range 4.6–111.1).^24^ Most recently, in a report on the LAANTERN registry, survival at 1, 3, 6, 12, and 24 months for the 34 patients with RN due to metastatic disease was 94.1%, 91.1%, 87.8%, 71.1%, and 71.1%, respectively. These outcomes appear favorable in this patient population given their limited treatment options.

Additional studies have compared LITT to other standard treatments for RN, such as surgery and bevacizumab. In a retrospective study by Hong et al. comparing 15 patients undergoing craniotomy versus 18 patients treated with LITT, the LITT group had similar 1- and 2-year PFS and OS compared to the craniotomy group, with no significant difference between the groups in the ability to taper off steroids or neurological outcomes.^17^ This study emphasizes the importance of cytoreduction in preventing both poor outcomes and long-term steroid usage. When compared to bevacizumab in retrospective study of 38 patients with symptomatic RN, LITT conferred a statistically significant improvement in median OS (24.8 vs 15.2 months, *P* = .003), trended toward longer time to local recurrence (12.1 vs 2.0 months, *P* = .091), and diminished lesion size at 1 year compared with the bevacizumab cohort.^22^ With the morbidity and therapeutic implications associated with open craniotomy and bevacizumab, a safe alternative intervention with minimal disruption in systemic treatments should offer significant advantages in these patients.

The extent of ablation has been shown to be predictive of response to LITT in multiple pathologies, most notably gliomas and brain metastases.^39^ However, the data are less clear regarding extent of ablation in LITT for RN. Of the available literature reporting on ablation volumes in LITT for RN, between 86% and 100% of the contrast-enhancing volume was treated.^19,22,27,38^ In Ahluwalia et al., the patients treated with LITT for RN had no local recurrence regardless of the extent of ablation.^27^ However, Luther et al. reported on their series of 20 patients with biopsy-proven RN that those who received subtotal ablations had high risk of local disease recurrence (HR 12.4, *P* = .004).^20^ Interestingly, patients that received radical ablations (>200% increase in pre-LITT lesion volume or >2 mm increase in pre-LITT lesion diameter) showed the most favorable PFS and OS, with no difference in post-LITT KPS and time to steroid freedom between ablation groups.^20^ Larger lesions may be more challenging to achieve a complete extent of ablation due to the technical considerations of LITT, however, Shao et al. reported that lesion size did not significantly affect OS or PFS in their series of 50 patients undergoing LITT for RN, with the caveat that all had lesions deemed size appropriate for the treatment.^39^ Given the likelihood that RN lesions may contain some component of recurrent tumor that was missed during biopsy, achieving maximal safe ablation is likely best practice until further studies are performed.

While steroid therapy is commonly employed in this patient population, recent data have shown the association of steroid use with worse clinical outcomes and counterproductive alterations to peripheral blood immune cell populations.^40^ As steroids also limit the efficacy of increasingly prevalent immunotherapies, the potential to employ LITT to limit long-term steroid use carries increased importance. Notably, in the early studies of LITT for RN, rapid and/or successful steroid wean was less common than it is today. In Rammo et al.’s report of 6 patients with brain metastases and post-SRS RN, 5 of the 6 patients were able to be weaned off of steroids by last follow-up, with 4 of them by the 4-week time point.^41^ In Swartz et al.’s series, 69.2% of patients tolerated a steroid wean post-LITT, at a mean time of 32 days (range 6–300) after treatment.^42^ Hernandez et al. reported that a majority of their 59 treated lesions were prescribed a 1–2 week taper, with 75% of patients requiring pre-LITT steroids successfully able to be weaned (and 87% of those not on steroids pre-LITT).^24^ Despite a significantly greater proportion of patients in the LITT group requiring steroid therapy for RN symptom control prior to treatment (68% for LITT compared to 46% for craniotomy) in Hong et al.’s study, the 1-month ability to wean steroids and rate of symptom improvement was statistically similar (35% vs 47% for ability to wean steroids, and 87% and 90% for symptom improvement for the LITT versus craniotomy groups, respectively).^17^ Understanding the role of LITT in reducing long-term steroid dependence in RN is an active area of investigation in the field, including by the authors, with 84% of LITT treated patients weaned off steroids post-treatment compared to 53% in a medical management cohort.^43^

The potential advantages of LITT in the treatment of RN are numerous: it is cytoreductive and minimally invasive, requiring only a small stab incision with short hospital stays; it can be combined with needle biopsy, allowing for definitive diagnosis; it has the ability to simultaneously treat lesions that are mixed with recurrent tumor and RN; it can treat deep-seated lesions inaccessible by open resection; it avoids the morbidity of craniotomy for previously irradiated lesions; and it allows for the rapid weaning of steroids and resumption of systemic therapies after the procedure.^17,19,24,39,43,44^ However, patient selection remains one of, if not the, essential question to guiding LITT therapy as each of the studies above are in some way limited by an initial screening for treatment candidacy as described in Figure 3. With this in mind, there is ample support for its use in the correct patient population with RN.

## LITT for Newly Diagnosed Metastatic Disease

While LITT for newly diagnosed glioma is a well-evidenced option for surgically inaccessible tumors, the treatment is less commonly used in newly diagnosed metastatic brain tumors given the efficacy and expanding treatment range of SRS.^45^ The literature describes few such cases, largely within broader studies that infrequently isolate the population for subgroup analysis. In their 2020 study investigating predictors of local control, Bastos et al. described 5 cases of newly diagnosed brain metastases comprising 6.6% of their total population, though this group was combined with the recurrent tumor data for much of the analyses.^18^ Salehi et al. described a further 2 cases out of 25 metastatic patients in which LITT was chosen as a first-line therapy due to the tumor location precluding safe resection, again without isolated analysis.^16^ Ashraf et al. reported 1 newly diagnosed metastasis in their series on LITT in the posterior fossa, and Rennert et al. identified 3 more cases representing 9% of their total brain metastases cohort.^29,46^ Lastly, a case report by Tan et al. describes the use of LITT as first-line treatment for a cholangiocarcinoma brain metastasis in the deep white matter of the cerebellum, chosen for its relative inaccessibility, without recurrence up to 16 months post-treatment.^47^ Without discrete data reporting from these cases it is difficult to draw many conclusions, though the sample provides a proof-of-concept for the treatment. At this time the applications of LITT for newly diagnosed brain metastases are limited, however future roles could develop particularly in locations that require radiation dose reduction or for more radioresistant histologies requiring cytoreduction in more difficult to access areas surgically.

## Limitations of LITT

As emphasized above, proper patient selection is essential to minimizing risks of adverse outcomes with the use of LITT. Heterogenous lesions with significant vascular or cystic components (along with those near ventricles) can render a challenge with respect to precise heat distribution, as the fluid “heat sinks” create asymmetrical ablation patterns.^48,49^ Typically, the maximum lesion diameter that can be treated with a single LITT trajectory is roughly 3 cm. Larger lesions may require multiple trajectories and have been associated with new or increased neurological deficits and worsened postoperative edema.^16,49,50^ Use of LITT near eloquent structures, such as language centers, motor cortex, or critical white matter tracts, is of particular interest for current and future studies. Sharma et al. examined the risk of postoperative motor deficits based on ablation overlap onto corticospinal tracts identified by diffusion tensor imaging (DTI). Their results emphasized the importance of minimizing even the slightest of ablation extension into these structures, with a proposed threshold of less than 0.046 cm^3^ overlap for the safest ablation.^51^ Our unpublished work on LITT near the motor cortex has shown that patients who survive out to 6 months have similar functional outcomes compared to craniotomy, with a trend toward greater benefit in pretreatment steroid responders.^52^

Identified risks of LITT include seizure, neurological deficits, bleeding, deep vein thrombosis, or infection across the existing studies, with perioperative complications and neurological deficits associated with prolonged hospital stays.^16,17,19,39,53^ The relationship between surgeon experience and complication rates is notable as well, as 1 study of 238 patients showed a significant improvement in rates of permanent postoperative motor deficits after increased institutional volume.^39^ This learning curve presents an important consideration in the adoption of this novel therapeutic technology. While LITT is an excellent option for many patients, it is not the right choice for every patient. Exploring nuances of patient selection based on morphology, location, and prognosis is essential to ensuring that the right patients receive the right treatment for their condition and goals.

## Future Directions

Currently, the administration and optimization of LITT are still in its early phases with tremendous opportunities for clarification and expansion. Looking to the future, compelling results showing a synergism between hyperthermic treatments, nanoparticles, and immunotherapeutics point to a direction that may further extend the scope of this approach in recurrent metastatic disease. Liu et al. highlighted this interaction in their preclinical study pairing plasmonic gold nanostars with laser ablation and checkpoint blockade immunotherapy, generating an effective antitumor response that evolved into long-term immunity resistant to tumor rechallenge.^54^ A broad range of evidence provides mechanistic support for immune modulation by hyperthermic cancer treatments.^55,56^ The TORCH clinical trial out of University of Florida is currently investigating the combination of LITT with the anti-PD-1 monoclonal antibody pembrolizumab in brain metastases, while several similar studies continue in primary brain tumors.^57–60^ Additional trials are exploring the use of adjuvant traditional chemo- and radiotherapies with LITT as well.^61–63^ The results of these studies, along with further investigation of interactions between LITT and SRS, may present radical new directions as emerging therapies are integrated for a multipronged approach to neuro-oncology.

## Conclusion

Over the past decade, LITT has emerged as an effective and safe minimally invasive treatment option for radiographically progressive metastatic lesions after SRS. Whether the etiology of radiographic progression is recurrent metastatic disease or RN, LITT has demonstrated equivalent or better clinical outcomes relative to alternative treatments in patients appropriate for the therapy. By expanding beyond those patients and lesions typically considered surgical candidates, the technology presents a novel option where there otherwise may be none. Continued evaluation of LITT and its integration with existing and emerging treatment modalities offers an avenue forward to identify new management strategies for the growing population with progressive intracranial metastatic disease.
